# Potential invasion of exotic ambrosia beetles *Xyleborus glabratus* and *Euwallacea* sp. in Mexico: A major threat for native and cultivated forest ecosystems

**DOI:** 10.1038/s41598-018-28517-4

**Published:** 2018-07-05

**Authors:** Andrés Lira-Noriega, Jorge Soberón, Julián Equihua

**Affiliations:** 1 CONACyT Research Fellow, Instituto de Ecología A. C., Red de Estudios Moleculares Avanzados, Carretera Antigua a Coatepec 351, El Haya, 91070 Xalapa, Veracruz Mexico; 2 Biodiversity Institute, University of Kansas, Lawrence, KS 66045 USA; 3 Comisión Nacional para el Conocimiento y Uso de la Biodiversidad, Liga Periférico - Insurgentes Sur 4903, Col. Parques del Pedregal, Delegación Tlalpan, 14010 D.F Mexico

## Abstract

We analyze the invasive potential of two Asian ambrosia beetles, *Xyleborus glabratus* and *Euwallacea* sp., into Mexico and the southern United States. The fungal symbionts of these beetles have been responsible for damage to trees of the family Lauraceae, including *Persea americana* and other non-cultivated tree species on both coasts of the United States. We estimate their potential threat using ecological niche modeling and spatial multi-criteria evaluation protocols to incorporate plant and beetle suitabilities as well as forest stress factors across Mexico. Mexico contains higher climatic and habitat suitability for *X. glabratus* than for *Euwallacea* sp. Within this country, the neotropical region is most vulnerable to invasion by both of these species. We also identify a corridor of potential invasion for *X. glabratus* along the Gulf of Mexico coast where most Lauraceae and native *Xyleborus* species are present; dispersal of either *X. glabratus* or *Euwallacea* sp. into this region would likely lead to major disease spread. However, the overall potential damage that these beetles can cause may be a function of how many reproductive hosts and how many other ambrosia beetles are present, as well as of their capacity to disperse. This work can also alert relevant managers and authorities regarding this threat.

## Introduction

Open economies and international trade promote the movement of exotic pests and diseases, creating a threat to agroecosystems and natural ecosystems^1,2^. As the problem of invasive species is a major cause of concern, many countries have created or adopted protocols for risk assessment and management^3^. In order to understand the risk of establishment of invasive species in new environments reliable modeling techniques are necessary^4,5^. One promising method for such risk assessment includes transferring ecological niche models of the species of concern onto landscapes where it has not yet arrived^6,7^. These models can be evaluated at a later stage with information about the biotic and abiotic factors that allow for the survival of the species for better assessing the invasion potential.

In 2002, the fungus *Raffaelea lauricola* T.C. Harr., Fraedrich & Aghayeva, the pathogen causing Laurel Wilt Disease (LWD), was introduced to Georgia, in the east coast of the United States^8^, causing major mortality in tree species of the Lauraceae family. This fungus is transmitted by the ambrosia beetle *Xyleborus glabratus* Eichhoff (Coleoptera: Curculionidae: Scolytinae;^9,10^), a species native to Asia that occurs over a broad geographic region from India, Bangladesh and Myanmar, through Japan, and Taiwan^11^. LWD induces mortality most frequently in plants that are under stress, although healthy individuals can also be attacked^9,12^. LWD has extirpated populations of Lauraceae species across southeastern United States, including *Persea borbonia* L. Spreng., *Persea palustris* Raf., *Lindera benzoin* (L.) Blume, *Sassafras albidum* (Nuttall) Nees, *Persea americana* Mill^9,13^, affecting coastal lowland ecosystems from North Carolina to Texas^14^. Impact of LWD on these tree species has turned it into one of the most threatening forest and agricultural diseases in North America^13^, since *P. americana* [avocado] is a major crop of Mexico and the United States. Although LWD is not the only disease capable of killing avocado trees, it acts most rapidly^15^.

Also of high concern for forest ecosystems in North America is the newly discovered *Euwallacea* sp. beetle (Polyphagous Shot Hole Borer; Coleoptera: Curculionidae: Scolytinae), which was originally identified as *E. fornicatus*^16^, a native to the region that stretches from Sri Lanka through Vietnam, China, Taiwan and the islands of Okinawa, Indonesia, Philippines and Papua New Guinea (CABI, 2018). *Euwallacea* sp. is associated with the fungus *Fusarium euwallaceae*, which is responsible for the emerging plant disease known as Fusarium dieback. *Euwallacea* sp. has proved to be polyphagous compared with *X. glabratus*. A recent report on the host range of the beetle-fungus complex based on two heavily infested botanical gardens in Los Angeles County indicated signs on 207 tree species representing 58 families^17^, including *P. americana*. An impact on avocado trees was demonstrated in 2012 in the United States^18^, as well as in Israel, where it has caused the death of large tracts of the Hass variety of *P. americana*^19^. Also, Fusarium dieback has been detected in San Diego, California, and has been found attacking trees in both urban and natural ecosystems in Tijuana, Mexico^20,21^. Should it continue spreading south, *Euwallacea* sp. has the potential to cause large impacts on Mexican biodiversity since many vulnerable plant families are abundantly represented further south in Mexico. These include Fagaceae (e.g., *Quercus agrifolia*, *Q. robus*), Platanaceae (e.g., *Platanus racemosa*), Magnoliaceae (e.g., *Magnolia grandiflora*), Euphorbiaceae (*Ricinus communis*) and Hamamelidaceae (e.g., *Liquidambar styraciflua*).

The arrival of these beetle-fungus complexes in Mexico could lead to huge natural and economic impacts on native and cultivated forest ecosystems. For example, the Mexican states of Chiapas, Oaxaca and Veracruz^22^ possesses 120 species of Lauraceae (90% of the Lauraceae diversity of the country). In Veracruz alone, 55 species of Lauraceae have been reported, 19 of them endemic. Mexico also has the largest avocado production in the world, representing a multibillion dollar industry^23^. According to official sources (SIAP; http://infosiap.siap.gob.mx/gobmx/datosAbiertos.php), in 2016 avocado was produced in 253 municipalities in 28 out of 32 states from Mexico. The total production for this year was 1,889,354 tons in 180,536 hectares. The value of this crop was approximately US $1.6 billion. Moreover, Mexico presents a large diversity of ambrosia beetle species both in regions where Lauraceae species are abundant as well as in avocado producing states, which could lead to the fungi rapidly spreading^24,25^.

Beetle dispersal into Mexico could take several possible routes, including commercial ports along borders with neighboring countries (particularly the United States) or along the coasts. Although dispersal for both beetle-fungus complexes is facilitated by wind^26^, this mode is potentially less important than human-facilitated events that move wood from beetle infested trees. Transportation of wood infected with *X. glabratus* has caused the spread of the disease over more than 700 km westward from the point of entry^27^. The current *X. glabratus*-*Raffaelea lauricola* complex present in the eastern United States and sudden appearances westwards up to Jasper and Hardin counties in eastern Texas make this state a priority for monitoring and evaluating suitable environmental conditions for this species complex, especially because the species has already bridged the Mississippi River, a natural barrier. Similarly, the dispersal of *Euwallacea* sp. into Baja California^20,21^ highlights the importance of monitoring along the border region.

Here we assess the potential risk of invasion and establishment of these two beetle-fungus complexes in Mexico. Predictions were developed using ecological niche modelling methods^28,29^ to estimate the suitable climates and habitats for their potential spread and establishment in Mexican ecosystems. Ecological niche models (ENMs) are commonly used to assess risk of the spread of non-native species^30^. We further refined this analysis using spatial multi-criteria evaluation (SMCE) tools^31^, with which we combined information on the suitable areas for the beetle species with the presence of relevant plant species, other ambrosia beetles and surrogates of stress in Mexican forests. We anticipate that through integrating different factors influencing the landscape suitability of *X. glabratus* and *Euwallacea* sp. would help to generate a framework to anticipate the establishment of these exotic species in areas where they had not yet arrived.

## Results

**Figure 1:**
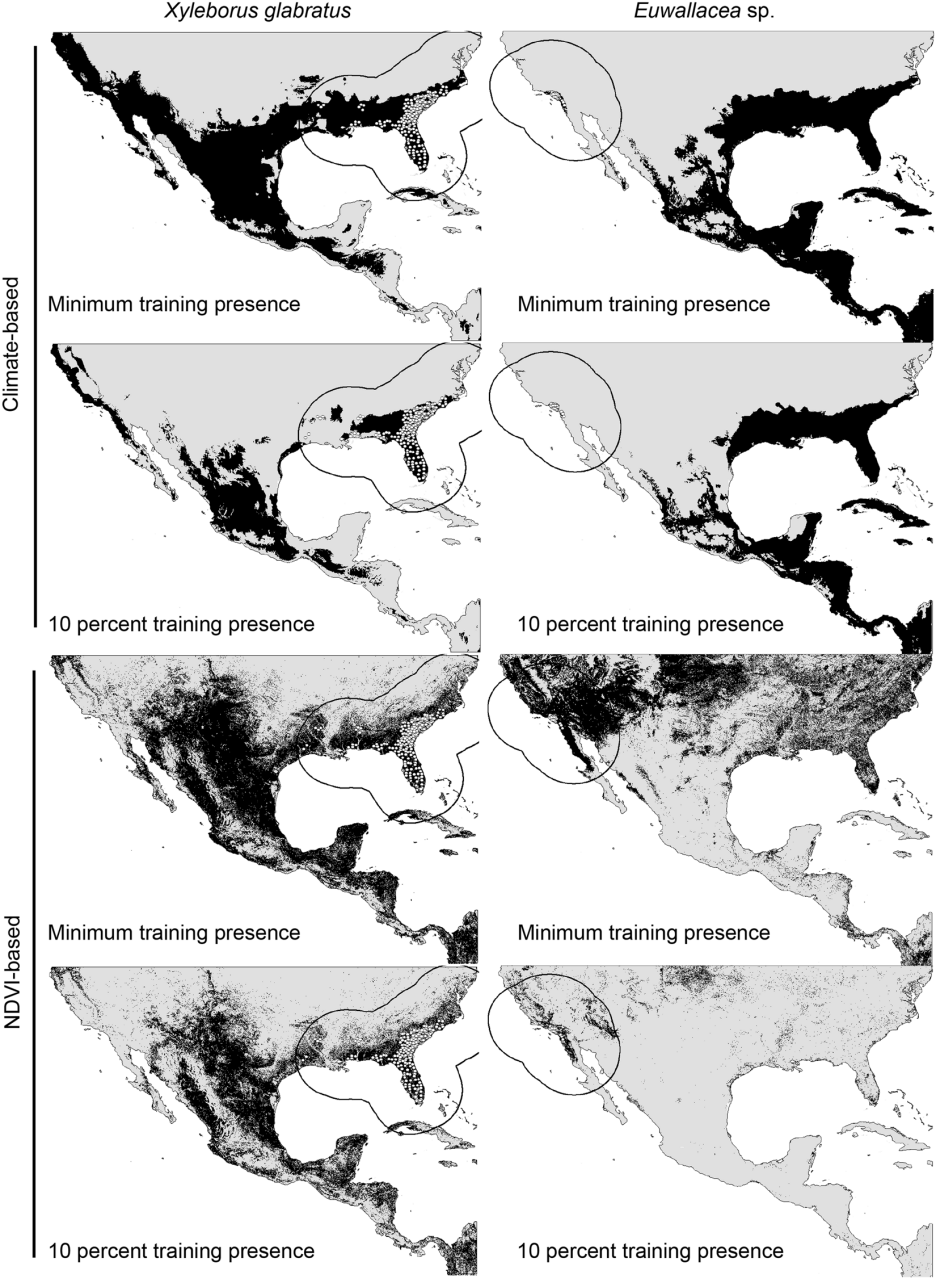
Suitabilities from ecological niche modeling for *X. glabratus* and *Euwallacea* sp. Areas of suitability (in black) for *X. glabratus* and *Euwallacea* sp from ecological niche model projections based on bioclimatic variables or Normalized Difference Vegetation Index (NDVI) and two thresholding levels for comparison. The related values were treated with GIS software (ArcGIS for Desktop, Software Version 10.2.2, http://resources.arcgis.com/en/home/) to generate the map in this figure.

All ENM models performed better than random expectations when tested against independent occurrence points, with all partial ROC AUC ratios above 1.0 and all p-values below 0.001 (Table S2). The models for each beetle species were used to estimate potential distributional areas in the southern United States and Mexico (Fig. 1). Model transfers were consistent for both thresholding levels; however, the climate-based transfer for *X. glabratus* and NDVI-based transfer for *Euwallacea* sp. at the 10^th^ percentile from the occurrence points showed a drastic reduction in suitable area compared with the minimum training presence thresholding (Fig. 1). A climate-based model for *X. glabratus* indicated suitable areas in southeastern United States where the species is currently established and along the Gulf of Mexico coast between Texas and Tamaulipas and southwards connecting at first with the dry and then with the humid forests; the Yucatan Peninsula was not identified as climatically suitable. Suitable climates for *Euwallacea* sp. were predicted precisely where the species is found today in California. The model transfer also predicted suitability in the southeastern portion of the United States and in Mexico in the moist forests along the Gulf of Mexico into the Yucatan Peninsula, and in Cuba and Central America.

An NDVI-based model for *X. glabratus* showed suitable areas in the southeastern United States where the species is currently established, and towards the Mississippi River, where suitability is truncated, to then through the entire south-central United States and in most of Mexico, with the exception of the western semiarid elevations and the Sonoran Desert. Suitable conditions extend along the Pacific coast, most of the temperate sierras and into the Yucatan Peninsula and Central America. NDVI-based models for *Euwallacea* sp. showed suitable areas in most of the xeric and dry forest of the western United States and northwestern Mexico near the known range of the species. A large extent of suitable habitat is also present across the United States with the minimum training presence threshold model; however, this area is reduced substantially in eastern United States in the model thresholded at 10 percent training presence.

**Figure 2:**
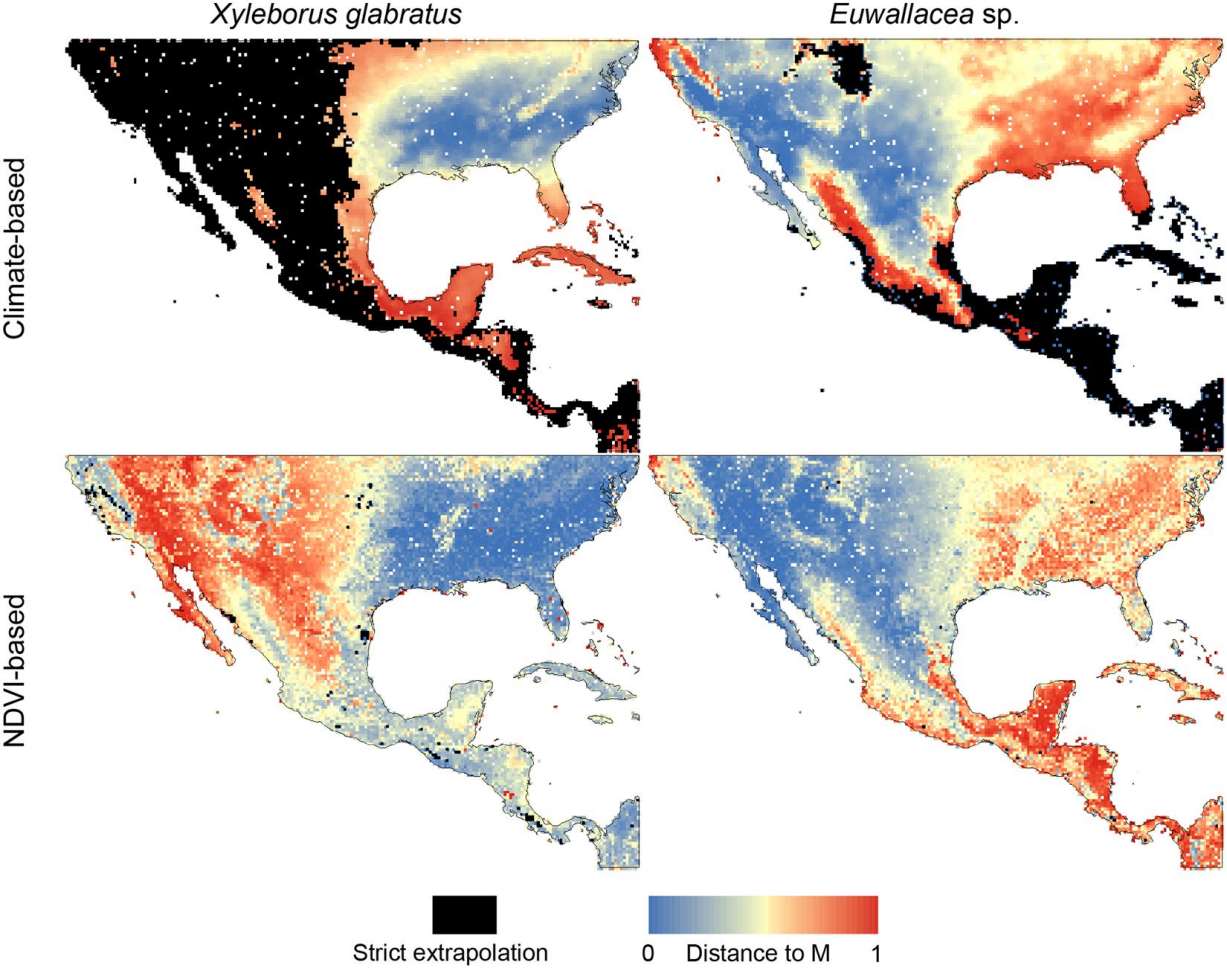
Environment similarity between model calibration regions and the projection extents. Mobility-oriented parity (MOP) assessment of environment similarity between model calibration regions (M) and the projection extent from each beetle species. Degree of similarity is in colors, and strict extrapolation is in black. The related values were imported and treated into GIS software (ArcGIS for Desktop, Software Version 10.2.2, http://resources.arcgis.com/en/home/) to generate the map in this figure.

MOP analyses revealed areas of the southern United States and Mexico that were not comparable with environments across the calibration regions for each beetle species; these areas differed depending on the type of environmental covariates used (Fig. 2). Environments showed strict extrapolation risk (i.e., variables outside the ranges in the training regions) for climatic combinations but rarely for NDVI combinations. For *X. glabratus*, these areas encompassed most of the area west of 99°W; the only similar regions in Mexico were along the Gulf Coast (including the Yucatan Peninsula). For *Euwallacea* sp., extrapolation risk only appeared around the Rocky Mountains and along both the Pacific and Gulf of Mexico coasts south of 24°N in Mexico and the Antilles. Indeed, most of the areas predicted to be suitable after the model was transferred with NDVI-based variables were validated for both species as non-extrapolative suggesting that we could use NDVI suitability predictions safely in the SMCE analysis.

**Figure 3:**
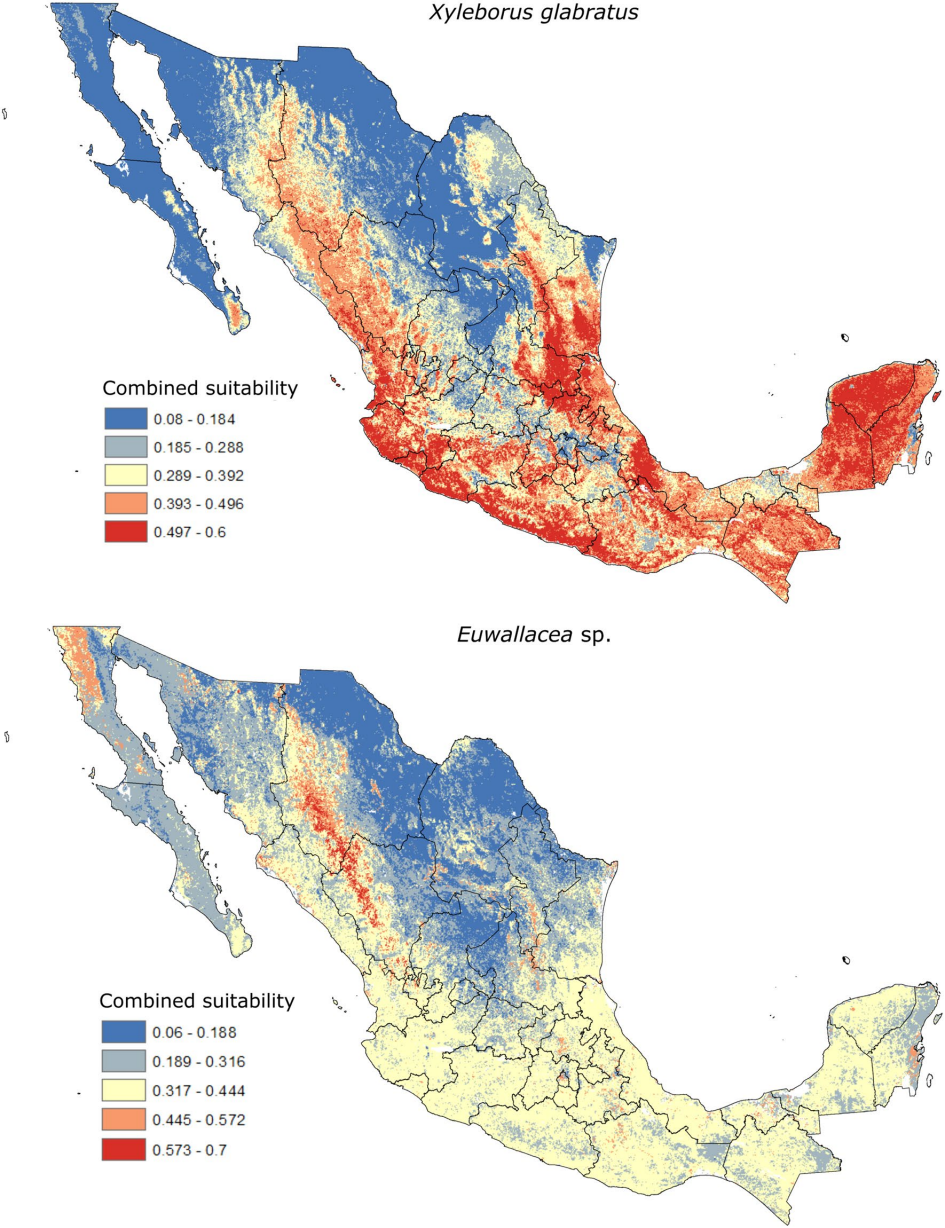
Suitability values for *X. glabratus* and *Euwallacea* sp. from spatial multi-criteria evaluations. The related values were imported and treated into GIS software (ArcGIS for Desktop, Software Version 10.2.2, http://resources.arcgis.com/en/home/) to generate the map in this figure.

The SMCE analysis indicated that the most suitable areas for *X. glabratus* in Mexico correspond to the tropical humid, tropical subhumid, and some temperate regions (Fig. 3). The most suitable areas in the humid tropical region corresponded to the Sierra Madre Oriental and the Gulf of Mexico in the states of Tamaulipas, San Luis Potosi, Veracruz, and southeast Mexico. In the tropical subhumid region, suitable areas were along the Pacific coast in the states of Nayarit, Jalisco, Colima, Michoacan, Guerrero, Mexico, Puebla, and Oaxaca. Temperate regions included suitability in parts of Sierra Madre Occidental in Sinaloa, Sonora, Durango, and Chihuahua, and the Mexican Transvolcanic Belt in Jalisco and Michoacan; other suitable areas were in San Luis Potosi, Oaxaca, and the isolated Cape region in the Peninsula of Baja California. All putative potential distribution areas of *X. glabratus* in Mexico coincided with areas with high diversity of Lauraceae and other ambrosia beetles, most of which are concentrated along the Sierra Madre Oriental and the southern sierras of Oaxaca and Chiapas. An SMCE for *Euwallacea* sp. in Mexico indicated lower suitability compared with *X. glabratus*, with the most suitable areas in the northern sierra of Baja California and around the temperate regions of the highland plateau of Chihuahua and Durango; intermediate suitability values corresponded to the potential distribution described for *X. glabratus* (Fig. 3).

**Figure 4:**
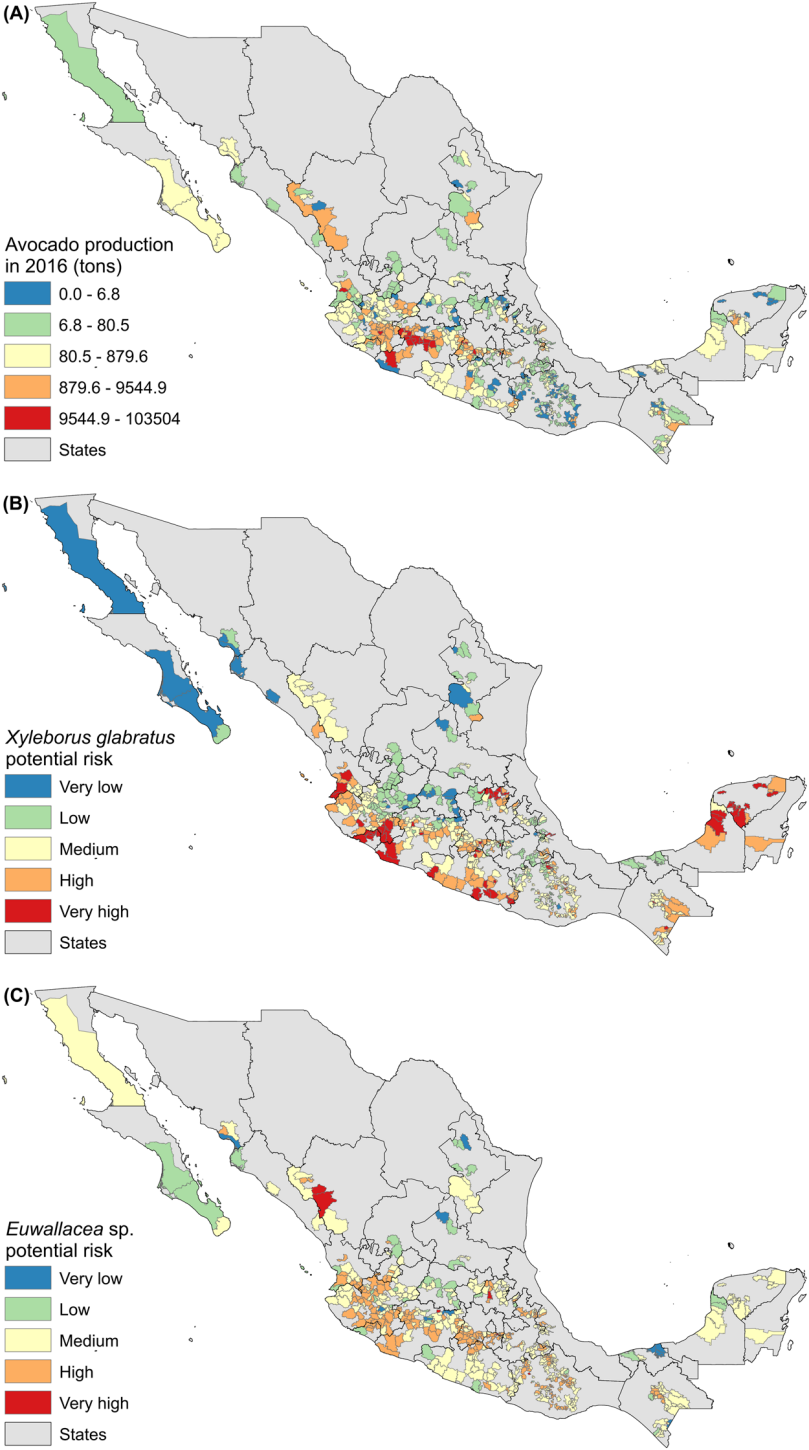
Map of municipalities producing avocado in Mexico indicating amount of production and risk of invasion by *X. glabratus* and *Euwallacea* sp. Values were imported and treated into GIS software (ArcGIS for Desktop, Software Version 10.2.2, http://resources.arcgis.com/en/home/) to generate the maps in this figure.

The largest production of avocado occurs in central Mexico, and mainly in Michoacan, but the risk of invasion for *X. glabratus* and *Euwallacea* sp. in the municipalities that are currently producing avocado in Mexico varied for each species (Fig. 4). For *X. glabratus* the areas of high and very high concern occurred in central west Mexico and the Peninsula of Yucatan, mainly in the states of Nayarit, Jalisto, Colima, Michoacan, Guerrero, Hidalgo, Queretaro, Chiapas, Campeche and Yucatan. For *Euwallacea* sp. these areas occurred in Durango, Nayarit, Jalisco, Colima, Michoacan, Mexico, Oaxaca, Hidalgo and Chiapas.

**Figure 5:**
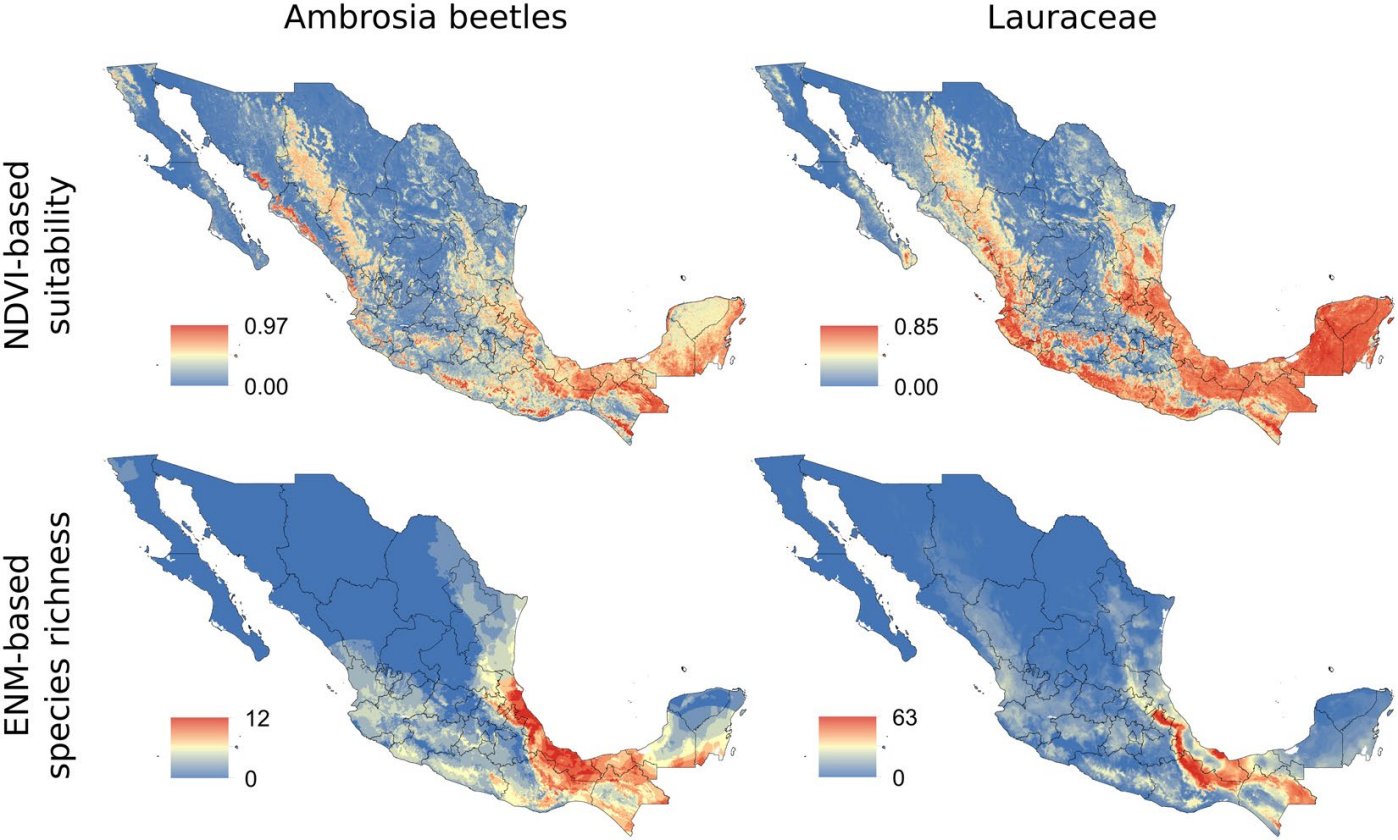
Suitabilities from ecological niche modeling for species of *Xyleborus* and Lauraceae based on Normalized Difference Vegetation Index (NDVI) and species richness for these groups of species derived from ecological niche models based on bioclimatic layers. The related values were treated with GIS software (ArcGIS for Desktop, Software Version 10.2.2, http://resources.arcgis.com/en/home/) to generate the maps in this figure.

Suitability predictions for species of Lauraceae and *Xyleborus* based on NDVI showed higher values across neotropical Mexico, but the largest species richness inferred from individual climate-based models for these groups of species concentrate in the southeastern part of the country (Fig. 5). Both the region of largest diversity of Lauraceae and *Xyleborus* appeared in Veracruz, Oaxaca, Campeche, Chiapas, and for Lauraceae it extends to southern Campeche and Quintana Roo (Fig. 5).

## Discussion

We found evidence for further invasive potential of *X. glabratus* and *Euwallacea* sp. in the southern United States and Mexico, where neither has reached a final extent. Although climate-based models indicated low suitability values in close proximity to current distributional areas, particularly for *Euwallacea* sp., suitable climate appears in other regions. NDVI-based ENM models indicate high suitability within their current distributional areas as well as more broadly across the areas we analyzed. These predictions suggest that establishing stringent sanitary measurements to contain further spread of these pests is imperative.

The NDVI-based ENM models have higher intrinsic resolution than the bioclimatic layers (these are interpolated from densities of climatic stations in the order of one in 10^3^ km^2^). Hence, the NDVI models provide a more detailed view of the potential for invasion, given the fact that they relate to land cover and seasonality and are good habitat proxies. Validation of the predictive capacity of NDVI models for these species should be considered a priority because NDVI models provide a more “pessimistic” view of potential species expansion.

The region of highest concern for the potential spread of *X. glabratus* is along the Gulf Coast, especially because (1) the beetle is already present in eastern Texas, (2) suitable climate and habitat are present towards the state of Tamaulipas and (3) these regions do not involve extrapolation in model transfer^32^. Because the suitabilities estimated with climate and NDVI for *Euwallacea* sp. are smaller and more restricted to its current distribution, the potential spread of this species is likely of lower concern; nevertheless, both climate and NDVI do not show extrapolation in the model transfer region, thus suggesting a wide area of suitable environments for this species to establish. We suggest that the model overfit observed for *Euwallacea* sp. results from a large number of occurrences in a very restricted area and a narrow combination of climates in this part of North America compared to its native range. This occurred despite our effort to rarefy the points used for modeling spatially and led to narrow predictions, a bias that has been reported previously (e.g.,^33^). More strikingly is that suitable climate for *Euwallacea* sp. was predicted in the southeastern United States. Although habitat in this region of the continent is dissimilar to this species’ calibration region, it corresponds to the area occupied by *X. glabratus* where at least two very important hosts (*Persea borbonia* and *Sassafras albida*) and cultivated trees (*P. americana*) are distributed.

No evident discontinuities appear in NDVI-based models for *X. glabratus*, with the exception of low or null habitat suitability along the Mississippi River Delta, which coincides with areas of lower climate suitability and low Lauraceae species presence, in particular the limits of the distribution of *P. borbonia* and *S. albida*, which are currently the two most important hosts for this species (T. A., pers. comm.).

The natural barrier represented by the Mississippi Delta has been breached, and the species is already in Texas, a serious cause for concern. In the case of *Euwallacea* sp., the NDVI-based model showed higher suitability closer to its current range, and the drastic reduction in suitability observed after applying the 10 percent threshold suggests that less suitable habitat could be expected southward. However, this outcome could be a result of the fact that *Euwallacea* sp. is currently present in a semiarid environment that only represents a small set of environmental combinations compared with its native distribution in Southeast Asia, indicating that the species has not yet arrived at its potential distribution limits in the New World^34^. Thus, potential species movement out of its current distribution (e.g., through wood transportation) to where it can meet more favorable conditions represents a major threat to native and cultivated ecosystems.

Both beetle species are associated with stressed or injured trees in their native ranges, as well as to healthy host trees in the invaded area (T. A., A. E., pers. comm.;^35^). The transmission of their symbiont fungus to other native or non-native beetle species could also contribute to the spread of the disease^24,25^. Using SMCE, we obtained a suitability value that incorporates the suitability prediction for each beetle, the presence of its hosts, and three forest stress-related factors reported for Mexico. The combined suitabilities from SMCE show large potential for successful invasion of the two beetle-fungus complexes in Mexico (Fig. 3). Although *X. glabratus* showed higher suitability values than *Euwallacea* sp., the polyphagous nature of *Euwallacea* sp. suggests that this species could expand its range much faster than *X. glabratus*, which is primarily restricted to species of Lauraceae. Moreover, damage could be greater if symbiont fungi are transferred to other beetle species (native or not) as the two target species colonize new regions, potentially causing faster dispersal^24,25^.

The risk of invasion of *X. glabratus* and *Euwallacea* sp. in municipalities that are currently producing avocado in Mexico indicates that several would suffer from the establishment of these species complexes. Highest vulnerability to *X. glabratus* occurs in the regions of central west Mexico and the Peninsula of Yucatan, mainly in Nayarit, Jalisto, Colima, Michoacan, Guerrero, Hidalgo, Queretaro, Chiapas, Campeche and Yucatan. The prediction for potential invasion of *Euwallacea* sp. shares a similar distribution but highest risk values are only in the states of Durango and Hidalgo, most likely due to its current distribution in California and Baja California in Mexico; however, the broad potential distribution for this species across Mexico implies a higher risk in several of the municipalities producing avocado. The municipalities with high and very high risk for both species were 331 in 22 states. This corresponds to an approximate production volume of 802,846 tons, approximately $635,881,718 US, according to information available for 2016 (SIAP; http://infosiap.siap.gob.mx/gobmx/datosAbiertos.php). Because of the importance of avocado production, many families depend now on this industry across the regions that produce it, which would be highly affected in case the plagues impacted this crop and its surrounding forest matrix.

The presence of the *Euwallacea* sp.-*Fusarium* sp. complex on the west coast of the United States and Tijuana in Mexico implies an urgent need to monitor this region given the huge impact that it has already caused in the United States. Additionally, the commercial routes from Tamaulipas and Veracruz to neighboring states of San Luis Potosi, Hidalgo, and Puebla, make the potential spread of the disease a substantially larger concern. These routes could eventually connect with the most important avocado croplands worldwide in Michoacan, Estado de Mexico, Nayarit, and Jalisco. More comprehensive efforts toward risk mapping and monitoring these species should include ports of entry, both maritime and terrestrial, and transportation of dead wood.

The analysis presented here encompasses environmental and ecological requirements that are considered important for the establishment of the two beetle species; however, little consideration has been given to their dispersal capacity. *X. glabratus* has been thoroughly documented to rapidly spread from the eastern United States westwards over the past 10 years *via* dead wood haulage. As mentioned in^35^, anecdotal estimates of dispersal rates for this species range from 30–100 km/yr to >200 km/yr with accidental human-mediated transport. Notably, accidental long-distance dispersal has already exceeded the predicted extent of *X. glabratus* predicted by^35^ based on a cost-distance model considering the distribution of redbay and sassafras, using a 54.8 km/yr dispersal rate. The intense commercial connection along the border between Mexico and the United States (which is particularly close to the present distributions of these two beetle-fungus complexes) must be considered in monitoring programs. Additionally, it is important to continue developing research to disentangle the environmental parameters influencing the successful establishment among different pathogens (fungi) and vectors (beetles), since biotic and abiotic requirement for different complexes of species and disease transmission may vary significantly. This information could be then used in monitoring programs that at the moment mostly look at either the beetles or the host trees, but rarely include the interaction of more factors.

Among several important factors that remain to be understood for better anticipating dispersal and establishment of these species are physiological limits of tolerance to adverse climatic conditions. Both beetle species can tolerate near freezing temperatures, and their development is apparently temperature-dependent^38,39^. In fact *X. glabratus* is the only ambrosia beetle for which supercooling point has been determined (−22 C in field conditions), and this may help to explain why this species and Laurel Wilt Disease could spread through eastern United States^40^. Also, cold-hardening may play an important response towards an increased capacity of the species to very cold temperatures^40^. The fact that these species are relatively cold-tolerant species may even increase the risk in lower latitudes where average temperatures are higher and there is less thermal seasonality compared to their current distributions in North America^41^. Apparently *X. glabratus* does not present diapause and females have been observed emerging during all the months of the year^40,42^, thus allowing the species to expand its range and invasion potential. This is particularly worrying if temperature increases as a consequence of global climate change, where winter minimum temperatures would not cause mortality of *X. glabratus*^41^, and may be the same scenario for *Euwallacea* sp. These and other physiological measures may also need to incorporate the thermal buffering capacity of bark and wood that offer protection from extreme weather oscillations^39,41^. Physiological related parameters could be used to develop mechanistic models, which could yield new insights on invasion potential for these beetle-fungus complexes^43,44^.

Our analysis is a first approximation in understanding the potential risk of invasion by these two beetle species. We emphasize that SMCE enables the use of different weights and standardizations of the factors used in the evaluation enabling different assessments in scenarios that require other factors or weights. Given the large extent and coarse resolution of the analysis, we assigned a relatively larger weight to the model’s suitability regarding beetles and hosts but less weight to forest stress factors. Nevertheless, results for both species also indicate high suitability values where forests present a larger proportion of dead trees and a variety of pests and where there is anthropogenic impact. Perhaps of highest concern are the geographic regions where both beetles’ suitabilities overlap with host species that are closely related phylogenetically; these represent more vulnerable hosts to the symbiont fungi, which increases the likelihood of infection^36,37^.

## Methods

### Occurrence data and environmental covariates

We retrieved occurrence localities to develop models for the beetles and for a suite of host plants, these include 24 species of *Xyleborus* and 89 of Lauraceae (see list of species in Table S1). Occurrences of *X. glabratus* in the United States were obtained from the literature and maps published by Bates *et al*.^27^ and by the Forest Service-USDA Forest Health Protection (^45^; kindly provided by the personnel from SENASICA in Mexico, see Acknowledgements). We retrieved data for *Euwallacea* sp. from the Polyphagous Shot Hole Borer/Fusarium Dieback Distribution Map (http://eskalenlab.ucr.edu/distribution.html).

From the Mexican National System of Biodiversity Information (SNIB; http://www.conabio.gob.mx/institucion/snib/doctos/acerca.html), the Global Biodiversity Information Facility (GBIF; http://www.gbif.org), and the Southwest Environmental Information Network (SEINet; http://swbiodiversity.org/seinet/), we retrieved all Mexican and United States localities for native Lauraceae and *Xyleborus* species, as well as other host species that have been affected by Fusarium dieback according to Eskalen *et al*.^17^. The species and number of occurrences are shown in Table S1.

To develop ecological niche models (ENMs), we used two types of covariates. First, four bioclimatic variables (maximum temperature of the warmest month, minimum temperature of the coldest month, precipitation of the wettest month, and precipitation of the driest month) at a spatial resolution of 2.5 minutes were used to estimate suitable climatic conditions for *X. glabratus* and *Euwallacea* sp.; these layers were downloaded from WorldClim (http://www.worldclim.org;^46^). We used this subset of the 19 bioclimatic variables included in WorldClim as a result of selecting best possible limiting factors for these beetle species with entomological experts (T. A. pers. comm.) and after conducting a jackknifing procedure in Maxent. Also, because our objective was to transfer predictions in space, it is advisable to minimize the overfitting effect induced by the use of many variables in ENM^28^.

A second source of covariates for ENM included Normalized Difference Vegetation Index (NDVI). NDVI is an index of photosynthetic activity related to vegetation cover, rain and temperature^47^. Because its resolution is relatively high (~1 km^2^ per pixel) and it depends on the presence of vegetation, NDVI can be regarded as a proxy for land cover type and state^47^. We summarized the variation of NDVI from the years 2002 to 2014 using principal component analysis (PCA) based on 50 standardized monthly NDVI layers of 1 km spatial resolution that presented no display problems or an excessive amount of clouds thus precluding their use. Variable standardization was performed with the ArcGIS Geomorphometry and Gradient Metrics toolbox^48^. NDVI layers were downloaded from the NASA Earthdata Search portal (https://earthdata.nasa.gov); MODIS Aqua monthly composite NDVI (MYD13A3) and were processed in batch mode using the MODIS Reprojection Tool (MRT). The data were converted from.hdf format to.tif format with a geographic WGS84 projection, mosaicked, and clipped to the following window: 41.34–4.98°N, 73.92–124.18°W. The PCA was computed with the ArcMap 10.1 Principal Components tool in the Spatial Analyst extension. We retained the first five principal components, which summarized 93% of the variance in the dataset.

### Ecological niche modeling

We estimated suitability indices using Maxent^49^. This algorithm has performed well in other analyses including biological invasions^50^, and in cases of partial sampling of the niche^51,52^. For *X. glabratus* and *Euwallacea* sp. we callibrated models based on climate and NDVI layers, as described above. These models allow us to identify what would be the most suitable regions for an invasion. For the Lauraceae and the other *Xyleborus* species included in SMCE protocol (see below), however, we used only NDVI covariates. Because these analyses aim to estimate the suitabilities for the entire set of species of either *Xyleborus* or Lauraceae, only one model was generated for the entire pool of records, for all the species in each of these taxonomic groups (Table S1). We interpret these as suitability maps for the genus *Xyleborus* and for the family Lauraceae as input for the multi-criteria evaluation.

To calculate diversity for the groups of species of *Xyleborus* and Lauraceae, we used modeled species by species, using only species with >3 and 15 occurrences, respectively. Such models were based on climate layers only and were converted to a binary (presence-absence) prediction based on a 10-percentile threshold, and finally were added in order to estimate Lauraceae’ and *Xyleborus*’ species richness. A summary of the species included in each of the modelling described here and their number of occurrences appears in Table S1 as part of the supplementary information.

ENMs are sensitive to the background region, with strong implications for niche modeling performance^53^; thus, we *a priori* designated model-calibrating regions based on biogeographic considerations. To this end, a 200-km buffer was placed around polygons that correspond to the ecoregions^54^ with occurrences for each species or set of species (i.e., for Lauraceae and *Xyleborus* species). The buffered regions were then used as calibration regions for Maxent (i.e., the areas from which to sample background points), and are assumed to incorporate a climatic and biogeographic relevant context in fitting the models^53^. The calibration regions for both *X. glabratus* and *Euwallacea* sp. incorporated all localities where each species has been found since its introduction in North America up to 8 June 2015. We then used these polygons to mask the environmental covariates that were used to calibrate each model, and models were then transferred onto environmental conditions across an extent encompassing most of United States, Mexico, and Central America.

We rarefied spatially the number of occurrences of Lauraceae and *Xyleborus* species by choosing points at a minimum distance of approximately ~5 km, considering that these species’ occurrence points were not highly spatially autocorrelated^55^. For the *Euwallacea* sp. and *X. glabratus* models, we used a selection of points at a minimum distance of approximately ~20 km, which is desirable when using Maxent and when localities are found very close to each other and concentrated in one small region^56^. All models were estimated using Maxent 3.3.3^49^ with 5 replicates and 25% testing points. The median of the raw output of each model was thresholded using a minimum training presence and the 10 percentile of training occurrence data to obtain binary predictions^52^.

When transferring ENM predictions, it is important to check for extrapolations outside the range of variables in the training regions or for very dissimilar climatic combinations. The similarity of environments between calibration areas and areas across the projection extent (i.e., most of North America) were assessed using mobility-oriented parity (MOP) analysis^32^. MOP refines the earlier multivariate environmental similarity surface (MESS) metrics implemented within Maxent to identify areas of simple transfer where interpretation is acceptable versus areas where extrapolation is occurring and interpretation should involve considerable caution^57^.

Prior to modeling for the two beetle complexes only using occurrences from the invaded ranges in North America, we tested that their niche spaces in Asia were recovered when models were calibrated in North America and transferred to the rest of the world. This step confirmed that there were no biases given the amount and location of occurrences and environmental predictors used in model calibration.

Ecological niche models were evaluated using the partial AUC approaches as implemented by Peterson *et al*.^58^, which enabled us to compare performance of each model versus random expectations. This procedure assesses the portions of the ROC curve that are relevant (i.e., within omission error tolerances) by calculating the ratio between the area under the curve for observed values against the area under the line of random discrimination; AUC ratios are expected to depart upwards from one if model performance is better than random. To this end, we randomly set aside testing points for each model (Table S2), and we multiplied each continuous model suitability prediction by 1,000 and converted the floating-point grid to an integer in R (R Development Core Team, 2015). With the modeled suitability values associated with each independent testing point, we then implemented partial AUC with custom scripts developed in R by N. Barve (https://github.com/narayanibarve). We ran 500 bootstrap simulations with *E* = 0.05 and 50% of the points resampled with replacement in each iteration of the bootstrap. Distributions of the randomized ratios were compared with Welch’s two-sample *t*-test to determine if the differences in the means of the random expectations to the partial AUC ratios were consistently larger than 1.

### Forest stress factors for spatial multi-criteria evaluation

We wished to use a spatial multi-criteria evaluation (SMCE), which requires the calculation of other variables associated with infestation by ambrosia beetles. In general, tree infections by ambrosia beetles are correlated with weak or stressed trees^59^, although exceptions exist (T. A., A. E., pers. comm.;^35,60^). Therefore, we compiled three variables that relate to forest stress for incorporation into the SMCE. Forest stress-related variables included the probability of plague insects on trees, the probability of standing dead trees, and an index of anthropogenic impacts on biodiversity. Maps of the probability of plague insects on trees and the probability of standing dead trees were generated as continuous layers with a spatial resolution of 1 km using Random Forest Classifiers (RFC) models based on information from the Mexican National Forestry and Soil Inventory (INFyS; http://www.cnf.gob.mx:8090/snif/portal/infys). INFyS measurements take place in cycles consisting in the sampling of 1/5 of ~22,000 plots that are distributed across Mexico every year. Every point is sampled once every 5 years. INFyS includes the collection of 152 variables on each plot, of which 39 are at the tree level including the ones we used^61^. For the layers used in this analysis, 22,025 plots from the 2004–2009 INFyS cycle were processed to serve as training data for two binary RFC models. Every plot with at least one count of a standing dead tree was labeled with the presence of standing dead trees (absence otherwise). A plot with at least one tree with insects was labeled as insects present (absence otherwise).

These two data sets were subsequently associated with several predictors available for Mexico. The first batch of predictors was derived from remote sensing information, including MODIS Terra 16-day vegetation indices at 1 km resolution (MOD1342) and MODIS 16-day Nadir BRDF-Adjusted Reflectance at 1 km resolution (MCD43B4). For each of the layers of these MODIS products, pixelwise composites were produced for the span of years 2004–2009 based on different functions applied to the image time series: mean, standard deviation, coefficient of variation, median, and the 20^th^, 35^th^, 65^th^, 80^th^, 95^th^ percentiles for both the dry (December-April) and wet (May-November) seasons, respectively. The second batch of predictors were climatic and topographic in nature: maximum, minimum and total precipitation monthly surfaces based on data comprising the period 1910–2009^62^, which were resampled to 1 km using the nearest neighbor method as they originally have a 30 arc second resolution^63^.

A total of 281 predictors entered RFC model training. RFC builds orthogonal classification trees^64^ using bagging, a meta-algorithm that generates new independent training sets by bootstrapping the original data^65,66^. RFC introduces additional variance to trees by combining bagging with the random subspace method^67^: the candidate variables for each split of each tree are restricted to a random sample of the features, in this case a sample size $\sqrt{281}$. Final models consisted of RFCs with 1,000 trees. Usually, individual tree results are aggregated with a majority prediction; however, instead of producing a hard classification by means of majority predictions, the proportion of predictions for the class “presence” was used to generate probability of presence maps. Taking a majority prediction (classification threshold = 50%), the presence of standing dead trees and presence of insects on trees models showed overall accuracies of 73% and 74%, respectively, estimated with bootstrapping (out-of-bag error). Data acquisition, preprocessing and model building for these layers were implemented in a combination of the R and Python programming languages.

Finally, the third forest stress-related factor corresponded to an index of anthropogenic impacts on biodiversity (MEXBIO;^68^). This summary is a spatial model of the relationships between pressure factors affecting biodiversity that follows the theoretical framework from the Global Biodiversity Model (GLOBIO3;^69,70^). Impacts of human activities on biodiversity were simulated by combining maps of the most relevant pressure factors including land use, infrastructure, fragmentation, and climate change.

### Spatial Multi-Criteria Evaluation

Spatial multi-criteria evaluations (SMCE) are conceived as a way to combine information between geographical information systems and multi-criteria decision analysis, and have proved useful in applied real management problem^71^. In the present case, ILWIS 3.8 (http://52north.org/communities/ilwis) was used to implement the SMCE. Here, we combined information from three sets of factors based on layers with a native resolution of 1 km: (1) beetle suitability derived from NDVI ecological niche models, (2) suitable areas of tree species that are vulnerable to these beetle species derived from NDVI ecological niche models, and (3) stress-related factors in forest ecosystems.

**Figure 6:**
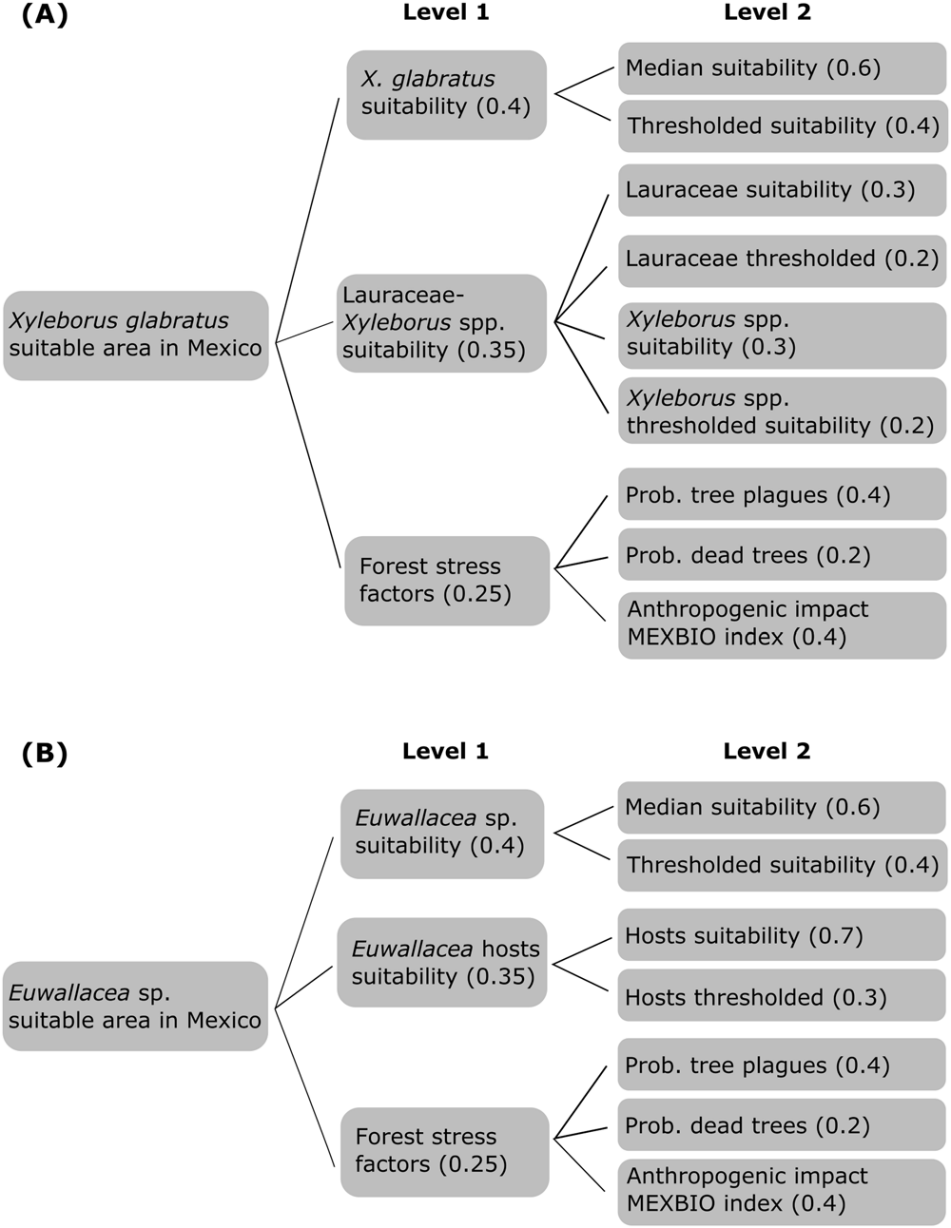
Decision trees for spatial multi-criteria evaluation. Decision trees used in the spatial multi-criteria evaluation for *X. glabratus* and *Euwallacea* sp. See Methods for details on variable weighting and standardization.

The multi-criteria decision tree for the suitability of each beetle species was formed by combining beetle and host suitabilities and forest stress factors in two nested levels (Fig. 6). The weights given to each factor represent their importance in the evaluation and were a suggestion based on the behavior of the system^72^. For *X. glabratus* suitability, level 1 corresponded to three sets of variables: (1) potential distribution (weight = 0.4), (2) vulnerable tree species suitability and other related beetle species suitability (0.35) and (3) forest and ecosystem stress factors (0.25). For *Euwallacea* sp., level 1 was the same as that for *X. glabratus* only without related beetle species. Level 2 corresponded to the nested sets of factors in level 1 as follows: beetle suitability estimated from ecological niche models (0.6) and beetle thresholded (binary) suitability (0.4), the suitability of vulnerable tree species (0.2), other related beetle species suitability (0.3), and their corresponding binary suitable area (0.2). We weighted and standardized each factor to account for their statistical and spatial distributions. Continuous suitability layers were standardized using a convex function (e.g., an exponential standardization for a raw Maxent score will correct for pixels in the projected ecological niche model with such small values as to make them irrelevant to the goal of the analysis), and thresholded suitabilities as well as forest stress-related factors were standardized using a goal function (i.e., a linear function that uses the minimum and maximum values from the raster layer). All variables were put on a Lambert Conformal Conic projection at their native spatial resolution of 1 km. In order to identify what would be the risk of invasion in avocado producing regions in Mexico, we summarized the SMCE final predictions for *X. glabratus* and *Euwallacea* sp. for the municipalities that according to agricultural official sources produced avocado in 2016 (SIAP; http://infosiap.siap.gob.mx/gobmx/datosAbiertos.php).

### Data availability statement

Upon publication of the manuscript, all data will be fully available without restriction.

## Electronic supplementary material

Supplementary Information

## References

[1] Pimentel D, Zuniga R, Morrison D (2005). Update on the environmental and economic costs associated with alien-invasive species in the United States. Ecol Econ.

[2] Tobin PC (2015). Ecological Consequences of Pathogen and Insect Invasions. Current Forestry Reports.

[3] Andersen MC, Adams H, Hope B, Powell M (2004). Risk Assessment for Invasive Species. Risk Analysis.

[4] Drake, J. A. & Environment, I. C. o. S. U. S. C. o. P. o. t. *Biological Invasions: A Global Perspective*. (Wiley, 1989).

[5] Peterson AT (2003). Predicting the geography of species’ invasions via ecological niche modeling. Quarterly Review of Biology.

[6] Peterson AT, Vieglais DA (2001). Predicting Species Invasions Using Ecological Niche Modeling: New Approaches from Bioinformatics Attack a Pressing ProblemA new approach to ecological niche modeling, based on new tools drawn from biodiversity informatics, is applied to the challenge of predicting potential species’ invasions. BioScience.

[7] Zhu G-P, Peterson AT (2017). Do consensus models outperform individual models? Transferability evaluations of diverse modeling approaches for an invasive moth. Biological Invasions.

[8] Rabaglia, R. *Xyleborus glabratu*s. *Exotic Forest Pest Information System for North Americ*a (2008).

[9] Fraedrich SW (2008). A fungal symbiont of the redbay ambrosia beetle causes a lethal wilt in redbay and other Lauraceae in the southeastern United States. Plant Disease.

[10] Mayfield AE (2008). Ability of the redbay ambrosia beetle (Coleoptera: Curculionidae: Scolytinae) to bore into young avocado (Lauraceae) plants and transmit the laurel wilt pathogen (*Raffaelea* sp.). Fla Entomol.

[11] Rabaglia RJ, Dole SA, Cognato AI (2006). Review of American Xyleborina (Coleoptera: Curculionidae: Scolytinae) Occurring North of Mexico, with an Illustrated Key. Annals of the Entomological Society of America.

[12] Hanula JL, Mayfield AE, Fraedrich SW, Rabaglia RJ (2008). Biology and host associations of redbay ambrosia beetle (Coleoptera: Curculionidae: Scolytinae), exotic vector of laurel wilt killing redbay trees in the southeastern United States. J Econ Entomol.

[13] Mayfield AE, Smith JA, Hughes M, Dreaden TJ (2008). First report of Laurel wilt disease caused by a *Raffaelea* sp. on avocado in Florida. Plant Disease.

[14] Gramling JM (2010). Potential effects of Laurel Wilt on the flora of North America. Southeast Nat.

[15] Ploetz RC (2012). Responses of avocado to laurel wilt, caused by *Raffaelea lauricola*. Plant Pathol.

[16] Stouthamer R (2017). Tracing the origin of a cryptic invader: phylogeography of the Euwallacea fornicatus (Coleoptera: Curculionidae: Scolytinae) species complex. Agr Forest Entomol.

[17] Eskalen A (2013). Host range of Fusarium Dieback and its ambrosia beetle (Coleoptera: Scolytinae) vector in southern California. Plant Disease.

[18] Eskalen, A. & Stouthamer, R. (California Avocado Commission and University of California, Riverside, 2012).

[19] Mendel Z (2012). An Asian ambrosia beetle Euwallacea fornicatus and its novel symbiotic fungus Fusarium sp pose a serious threat to the Israeli avocado industry. Phytoparasitica.

[20] Equihua-Martínez A (2016). New host association between *Euwallaceae* sp. (Coleoptera: Curculionidae: Scolytinae) and *Casuarina cunninghamiana* Miq. (Casuarinaceae) in Tijuana, Baja California Norte, Mexico. Folia Entomológica Mexicana (nueva serie).

[21] García-Avila CdJ (2016). First report of *Euwallacea* nr. *fornicatus* (Coleoptera: Curculionidae) in Mexico. Fla Entomol.

[22] Lorea Hernández FG (2002). La familia Lauraceae en el sur de México: Diversidad, distribución y estado de conservación. Boletín de la Sociedad Botánica de México.

[23] APEAM. *Association of Mexican Avocado Growers, Packagers and Exporters*., http://www.apeamac.com (2014).

[24] Carrillo D (2014). Lateral transfer of a phytopathogenic symbiont among native and exotic ambrosia beetles. Plant Pathol.

[25] Castrejón-Antonio JE (2017). Especies de Xyleborus (Coleoptera: Curculionidae: Scolytinae) asociados a huertos de aguacate en Colima, México. Acta zoológica mexicana.

[26] Salom SM, McLean JA (1991). Environmental influences on dispersal of *Tryopodendron lineatum* (Coleoptera: Scolytidae). Environ Entomol.

[27] Bates, C. *et al*. (Forest Service-USDA, 2015).

[28] Peterson, A. T. *et al*. *Ecological niches and geographic distributions*. (Princeton University Press, 2011).

[29] Václavík T, Meentemeyer RK (2009). Invasive species distribution modeling (iSDM): Are absence data and dispersal constraints needed to predict actual distributions?. Ecological Modelling.

[30] Escobar LE, Qiao H, Phelps NBD, Wagner CK, Larkin DJ (2016). Realized niche shift associated with the Eurasian charophyte Nitellopsis obtusa becoming invasive in North America. Scientific Reports.

[31] Malczewski, J. *GIS and Multicriteria Decision Analysis*. 392 (Wiley, 1999).

[32] Owens HL (2013). Constraints on interpretation of ecological niche models by limited environmental ranges on calibration areas. Ecological Modelling.

[33] Lira-Noriega A, Soberón J, Miller CP (2013). Process-based and correlative modeling of Desert Mistletoe distribution: A multiscalar approach. Ecosphere.

[34] CABI. *Invasive Species Compendium*, http://www.cabi.org/cpc/ (2017).

[35] Koch FH, Smith WD (2008). Spatio-temporal analysis of *Xyleborus glabratus* (Coleoptera: Circulionidae: Scolytinae) invasion in eastern US forests. Environ Entomol.

[36] Gilbert GS, Magarey R, Suiter K, Webb CO (2012). Evolutionary tools for phytosanitary risk analysis: Phylogenetic signal as a predictor of host range of plant pests and pathogens. Evolutionary Applications.

[37] Robles-Fernández, Á. L. & Lira-Noriega, A. Combining Phylogenetic and Occurrence Information for Risk Assessment of Pest and Pathogen Interactions with HostPlants. *Frontiers in Applied Mathematics and Statistic*s **3**, 10.3389/fams.2017.00017 (2017).

[38] Brar GS, Capinera JL, Kendra PE, Smith JA, Peña JE (2015). Temperature-Dependent Development of Xyleborus glabratus (Coleoptera: Curculionidae: Scolytinae). Fla Entomol.

[39] Cooperband MF (2016). Biology of two members of the Euwallacea fornicatus species complex (Coleoptera: Curculionidae: Scolytinae), recently invasive in the USA, reared on an ambrosia beetle artificial diet. Agr Forest Entomol.

[40] Formby JP, Krishnan N, Riggins JJ (2013). Supercooling in the Redbay Ambrosia Beetle (Coleoptera: Curculionidae). Fla Entomol.

[41] Formby JP (2018). Cold tolerance and invasive potential of the redbay ambrosia beetle (Xyleborus glabratus) in the eastern United States. Biological Invasions.

[42] Maner ML, Hanula JL, Braman SK (2013). Gallery Productivity, Emergence, and Flight Activity of the Redbay Ambrosia Beetle (Coleoptera: Curculionidae: Scolytinae). Environ Entomol.

[43] Kearney M, Porter WP, Williams C, Ritchie S, Hoffmann AA (2009). Integrating biophysical models and evolutionary theory to predict climatic impacts on species’ ranges: the dengue mosquito Aedes aegypti in Australia. Functional Ecology.

[44] Maino, J. L. & Kearney, M. R. Testing mechanistic models of growth in insects. *Proceedings of the Royal Society B: Biological Sciences* **282**, 10.1098/rspb.2015.1973 (2015). 10.1098/rspb.2015.1973 PMC4685816 26609084

[45] Forest *et al*. (Georgia Forestry Commission, South Carolina Forestry Commission, and USFS-Forest Health Protection, Asheville Field Office, 2008).

[46] Hijmans RJ, Cameron SE, Parra JL, Jones PG, Jarvis A (2005). Very high resolution interpolated climate surfaces for global land areas. International Journal of Climatology.

[47] Gates, D. M. *Biophysical Ecology*. 611 (Springer-Verlag, 1980).

[48] An AGIS Toolbox for Surface Gradient and Geomorphometric Modeling v. 2.0 (http://evansmurphy.wix.com/evansspatial, 2014).

[49] Phillips SJ, Anderson RP, Schapire RE (2006). Maximum entropy modeling of species geographic distributions. Ecological Modelling.

[50] West AM, Kumar S, Brown CS, Stohlgren TJ, Bromberg J (2016). Field validation of an invasive species Maxent model. Ecological Informatics.

[51] Elith J (2006). Novel methods improve prediction of species’ distributions from occurrence data. Ecography.

[52] Pearson RG, Raxworthy CJ, Nakamura M, Peterson AT (2007). Predicting species distributions from small numbers of occurrence records: a test case using cryptic geckos in Madagascar. Journal of Biogeography.

[53] Barve N (2011). The crucial role of the accessible area in ecological niche modeling and species distribution modeling. Ecological Modelling.

[54] Olson D (2001). Terrestrial ecoregions of the world: a new map of life on Earth. BioScience.

[55] Fourcade Y, Engler JO, Rodder D, Secondi J (2014). Mapping species distributions with MAXENT using a geographically biased sample of presence data: A performance assessment of methods for correcting sampling bias. PLoS ONE.

[56] Kramer-Schadt S (2013). The importance of correcting for sampling bias in MaxEnt species distribution models. Diversity and Distributions.

[57] Elith J, Kearney M, Phillips S (2010). The art of modelling range-shifting species. Methods Ecol Evol.

[58] Peterson AT, Papes M, Soberón J (2008). Rethinking receiver operating characteristic analysis applications in ecological niche modeling. Ecological Modelling.

[59] Blanchette, R. A. & Biggs, A. R. *Defense mechanisms of woody plants against fungi*. (Springer-Verlag Berlin Heidelberg, 1992).

[60] Peña JE (2012). Susceptibility of *Persea* spp. and other Lauraceae to attack by redbay ambrosia beetle, *Xyleborus glabratus* (Coleoptera: Curculionidae: Scolytinae). Fla Entomol.

[61] CONAFOR. *Manual y procedimientos para el muestreo de campo. Re-muestreo**2010*. (Inventario Nacional Forestal y de Suelos, Comisión Nacional Forestal, 2010).

[62] Cuervo-Robayo AP (2014). An update of high-resolution monthly climate surfaces for Mexico. International Journal of Climatology.

[63] INEGI. (Instituto Nacional de Estadística Geografía e Informática, Mexico, 1994).

[64] Breiman, L., Friedman, J., Olshen, R. & Stone, C. *Classification and regression trees*. (Chapman & Hall/CRC, 1984).

[65] Breiman L (1996). Bagging predictors. Mach Learn.

[66] Breiman L (2001). Random forests. Mach Learn.

[67] Ho TK (1998). The random subspace method for constructing decision forests. Ieee T Pattern Anal.

[68] CONABIO. (Comisión Nacional para el Conocimiento y Uso de la Biodiversidad, México, 2009).

[69] Alkemade R (2009). GLOBIO3: A framework to investigate options for reducing global terrestrial biodiversity loss. Ecosystems.

[70] GLOBIO. Global methodology for mapping human impacts on the biosphere. C. Nellemann, Kullerud, L., Vistnets, I., Forbes, B.C., Foresman, T., Husby, E., Kofinas, G.P., Kaltenborn, B.P., Rouaud, J., Magomedova, M., Bobiwash, R., Lambrechts, C., Shei, P.J., Tveitdal, S., Gron, O., Larsen, T.S. (UNEGP/DEWA/TR, 2001).

[71] Malczewski, J. *GIS and Multicriteria Decision Analysis*. (Wiley, 1999).

[72] van der Merwe JH, Lohrentz G (2001). Demarcating coastal vegetation buffers with multicriteria evaluation and GIS at Saldanha Bay, South Africa. AMBIO.

