# Supplementary material for: Potential invasion of exotic ambrosia beetles Xyleborus glabratus and Euwallacea sp. in Mexico: A major threat for native and cultivated forest ecosystems
Source: Sci Rep. 2018 Jul 5;8:10179. doi: 10.1038/s41598-018-28517-4 (PMC6033885; doi:10.1038/s41598-018-28517-4)
Supplement: Supplementary file 1 — Supplementary Information [file 41598_2018_28517_MOESM1_ESM.pdf]

Supplementary Information

Potential invasion of exotic ambrosia beetles *Xyleborus glabratus* and *Euwallacea* sp. in Mexico: A major threat for native and cultivated forest ecosystems

Andrés Lira-Noriega[1*], Jorge Soberón[2], Julián Equihua[3]

[1] CONACyT Research Fellow, Instituto de Ecología A. C., Red de Estudios Moleculares Avanzados, Carretera Antigua a Coatepec 351, El Haya, 91070 Xalapa, Veracruz, México.

[2] Biodiversity Institute, University of Kansas, Lawrence, KS 66045, USA.

[3] Comisión Nacional para el Conocimiento y Uso de la Biodiversidad, Liga Periférico - Insurgentes Sur 4903, Col. Parques del Pedregal, Delegación Tlalpan, 14010, México, D.F.

* Corresponding author

E-mail: aliranoriega@gmail.com

Work telephone number: +52(228) 842 1800 ext. 3602

Work fax number: +52(228) 818 780

Supplementary Table S1. List of species used to generate ecological niche models.

Main species and SMCE layers	Number of occurrences
Xyleborus glabratus	366
Euwallacea sp.	504
Xyleborus sp.	10117
Lauraceae sp.	8876
Hosts *Euwallacea* sp.	27561

Xyleborus sp.	
Xyleborus affinis	89
Xyleborus atratus	10
Xyleborus californicus	14
Xyleborus celsus	5
Xyleborus declivis	6
Xyleborus ferrugineus	68
Xyleborus horridatus	4
Xyleborus horridus	10
Xyleborus imbellis	4
Xyleborus intrusus	31
Xyleborus macer	10
Xyleborus posticus	16
Xyleborus princeps	20
Xyleborus pseudotenuis	5
Xyleborus schedli	4
Xyleborus schildi	4
Xyleborus scopulorum	5
Xyleborus sharpi	3
Xyleborus spathipennis	18
Xyleborus spinulosus	5
Xyleborus swezeyi	4
Xyleborus vespatorius	3
Xyleborus volvulus	64
Xyleborus xylographus	5

Lauraceae >15 occurrences in extent	
Aiouea inconspicua	23
Beilschmiedia hondurensis	23
Beilschmiedia mexicana	48
Beilschmiedia ovalis	38
Beilschmiedia pendula	60

Beilschmiedia riparia	25
Cassytha filiformis	163
Cinnamomum areolatum	15
Cinnamomum camphora	25
Cinnamomum effusum	54
Cinnamomum hartmannii	53
Cinnamomum neurophyllum	44
Cinnamomum pachypodum	90
Cinnamomum padiforme	15
Cinnamomum salicifolium	32
Cinnamomum triplinerve	235
Cinnamomum verum	23
Licaria capitata	77
Licaria cervantesii	20
Licaria excelsa	54
Licaria misantlae	59
Licaria peckii	72
Licaria triandra	65
Lindera benzoin	224
Litsea glaucescens	467
Litsea muelleri	22
Litsea neesiana	18
Litsea pringlei	17
Nectandra ambigens	34
Nectandra cissiflora	18
Nectandra coriacea	145
Nectandra cuspidata	73
Nectandra globosa	33
Nectandra hihua	121
Nectandra lineata	106
Nectandra loesenerii	28
Nectandra longicaudata	52
Nectandra lundellii	41
Nectandra martinicensis	93
Nectandra membranacea	201
Nectandra nitida	136
Nectandra purpurea	62
Nectandra reticulata	118
Nectandra salicifolia	658
Nectandra salicina	89
Nectandra sanguinea	29

Nectandra turbacensis	65
Ocotea acuminatissima	35
Ocotea betazensis	21
Ocotea botrantha	18
Ocotea bourgeauviana	17
Ocotea cernua	270
Ocotea dendrodaphne	232
Ocotea effusa	44
Ocotea helicterifolia	156
Ocotea heydeana	17
Ocotea klotzschiana	42
Ocotea laetevirens	167
Ocotea leucoxylon	199
Ocotea macrophylla	75
Ocotea oblonga	29
Ocotea psychotrioides	59
Ocotea puberula	52
Ocotea purpurea	46
Ocotea rovirosae	15
Ocotea rubriflora	31
Ocotea sinuata	61
Ocotea sprucei	20
Ocotea stenoneura	44
Ocotea tampicensis	32
Ocotea uxpanapana	34
Ocotea vanderwerffii	20
Ocotea veraguensis	266
Ocotea verticillata	15
Persea americana	586
Persea borbonia	26
Persea donnell-smithii	30
Persea hintonii	27
Persea liebmannii	84
Persea longipes	19
Persea palustris	32
Persea podadenia	40
Persea purpusii	21
Persea schiedeana	117
Persea sprucei	19
Persea vesticula	39
Phoebe tampicensis	25

Sassafras albidum	270
Umbellularia californica	737
**Lauraceae <15 occurrences in extent**	
Acrodiclidium glabrum	2
Acrodiclidium lucidum	2
Acrodiclidium misantlae	1
Androglandula tennessensis	1
Beilschmiedia anay	13
Beilschmiedia angustielliptica	2
Beilschmiedia manantlanensis	7
Beilschmiedia ovalioides	6
Beilschmiedia yaanica	1
Cinnamomum acrodromum	3
Cinnamomum amplexicaule	3
Cinnamomum appelianum	1
Cinnamomum arsenei	4
Cinnamomum blumei	2
Cinnamomum bractefoliaceum	14
Cinnamomum breedlovii	4
Cinnamomum cassia	2
Cinnamomum chiapense	3
Cinnamomum concinnum	1
Cinnamomum dilleri	10
Cinnamomum glossophyllum	1
Cinnamomum grisebachii	9
Cinnamomum leptophyllum	1
Cinnamomum sleumeri	3
Cinnamomum spectabile	1
Cinnamomum spurium	8
Cinnamomum validinerve	1
Cinnamomum vanderwerffii	1
Cinnamomum vanderwerfii	1
Cinnamomum velveti	2
Cinnamomum zapatae	12
Cryptocarya praesamarensis	1
Endiandra latifolia	7
Laurinoxylon pulchrum	1
Laurophyllum fremontensis	2
Laurophyllum litseaefolia	2
Laurus angusta	2

Laurus californica	1
Laurus cervantesii	1
Laurus grandis	1
Laurus ligi	2
Laurus longipes	1
Laurus machilus	13
Laurus montana	1
Laurus parvifolia	2
Laurus perdita	1
Laurus primigenia	3
Laurus princeps	1
Laurus tamala	4
Licaria alata	3
Licaria campechiana	13
Licaria caudata	9
Licaria chinanteca	2
Licaria coriacea	4
Licaria glaberrima	1
Licaria guatemalensis	6
Licaria ibarrae	2
Licaria nayaritensis	1
Licaria urceolata	4
Licaria velutina	7
Lindera angustifolia	1
Lindera kariensis	1
Lindera melissifolia	11
Lindera obtusa	9
Litsea aestivalis	4
Litsea glauca	1
Litsea novoleontis	3
Litsea orizabae	2
Litsea pallens	2
Litsea parvifolia	13
Litsea pedicellata	4
Litsea schaffneri	3
Malapoenna cuneata	1
Malapoenna lamarensis	1
Malapoenna weediana	1
Mespilodaphne moschata	1
Misanteca capitata	3
Misanteca peckii	3

Mocinnodaphne cinnamomoidea	1
Mutisiopersea liebmannii	1
Nectandra chiapensis	1
Nectandra colorata	4
Nectandra glabrescens	13
Nectandra heydeana	6
Nectandra leucocome	4
Nectandra matudai	1
Nectandra perdubia	10
Nectandra rubriflora	7
Nectandra rudis	6
Nectandra savannarum	1
Nectandra sinuata	12
Nectandra tabascensis	2
Nectandra tonii	1
Neolitsea lata	2
Ocotea bernoulliana	10
Ocotea campechiana	1
Ocotea candidovillosa	6
Ocotea chiapensis	2
Ocotea congregata	5
Ocotea corrugata	2
Ocotea heribertoi	1
Ocotea iridescens	3
Ocotea lundellii	3
Ocotea magnifolia	6
Ocotea matudae	3
Ocotea obtusifolia	2
Ocotea ovandensis	1
Ocotea parvula	9
Ocotea platyphylla	7
Ocotea pyramidata	1
Ocotea salvinii	4
Ocotea sarcodes	4
Ocotea sauroderma	3
Ocotea standleyi	8
Ocotea subalata	2
Ocotea tonii	2
Ocotea truncata	6
Oreodaphne benthamiana	1
Oreodaphne sieberi	1

Persea albida	8
Persea aurata	3
Persea brevipetiolata	2
Persea chamissonis	10
Persea cinerascens	6
Persea coriacea	4
Persea domingensis	2
Persea flavifolia	4
Persea floccosa	3
Persea humilis	3
Persea liebmanni	6
Persea matudae	2
Persea obscura	2
Persea pachypoda	3
Persea parviflora	1
Persea parvifolia	7
Persea praelingue	7
Persea pseudocarolinensis	11
Persea rufescens	10
Persea standleyi	4
Persea steyermarkii	5
Persea veraguensis	3
Perseanthus crossmanensis	1
Phoebe acuminatissima	5
Phoebe amplexicaulis	1
Phoebe angustata	1
Phoebe arsenei	2
Phoebe barbeyana	1
Phoebe benthamiana	1
Phoebe bourgeauviana	6
Phoebe chinantecorum	3
Phoebe effusa	3
Phoebe ehrenbergii	3
Phoebe gentlei	6
Phoebe helicterifolia	12
Phoebe longicaudata	1
Phoebe obtusata	1
Phoebe pachypoda	5
Phoebe padiformis	2
Phoebe pallescens	3
Phoebe platyphylla	1

Phoebe psychotrioides	4
Phoebe salicifolia	1
Phoebe salvinii	2
Phoebe siltepecana	1
Tetranthera glaucescens	2
Umbellularia parvifolia	1

Euwallacea hosts from Akif et al. (2013)	
Acer buergerianum	5
Acer macrophyllum	920
Acer negundo	1106
Acer palmatum	19
Acer rubrum	308
Albizia julibrissin	121
Alnus rhombifolia	881
Beaucarnea recurvata	35
Bocconia arborea	187
Brahea armata	24
Bursera fagaroides	12
Bursera hindsiana	141
Calocedrus decurrens	717
Carpinus caroliniana	469
Carya illinoinensis	187
Casimiroa edulis	205
Castanospermum australe	2
Catalpa speciosa	146
Cercidium floridum	485
Cercidium sonorae	31
Chiranthodendron pentadactylon	44
Clethra macrophylla	76
Corymbia ficifolia	4
Ehretia austin-smithii	3
Ehretia latifolia	183
Erythrina coralloides	51
Ficus maxima	460
Fouquieria macdougalii	50
Fraxinus uhdei	229
Fraxinus velutina	584
Garrya wrightii	151
Handroanthus impetiginosus	7
Hauya microcerata	5

Heliocarpus donnellsmithii	198
Ilex cornuta	17
Jatropha cinerea	111
Lippia torresii	21
Lippia umbellata	200
Liquidambar styraciflua	465
Liriodendron tulipifera	272
Magnolia guatemalensis	8
Montanoa guatemalensis	53
Nyssa sylvatica	240
Olmediella betschleriana	35
Ostrya virginiana	455
Parkinsonia aculeata	364
Persea americana	597
Pinus douglasiana	55
Pithecellobium dulce	507
Platanus mexicana	119
Platanus occidentalis	334
Platanus racemosa	745
Platanus wrightii	118
Populus fremontii	1028
Populus trichocarpa	607
Prosopis articulata	62
Prunus caroliniana	59
Pseudobombax ellipticum	183
Quercus agrifolia	1455
Quercus alba	248
Quercus chrysolepis	1662
Quercus emoryi	190
Quercus engelmannii	439
Quercus lobata	760
Quercus macrocarpa	247
Quercus mexicana	132
Quercus robur	2
Quercus salicifolia	4
Quercus virginiana	98
Ricinus communis	757
Salix babylonica	53
Salix gooddingii	1079
Salix laevigata	1278
Sambucus nigra	1109

Sequoia sempervirens	618
Sophora secundiflora	146
Tabebuia impetiginosa	169
Taxodium mucronatum	310
Thevetia thevetioides	63
Tilia americana	294
Ulmus alata	125
Ulmus americana	386
Umbellularia californica	1059
Ungnadia speciosa	75
Washingtonia filifera	95
Wisteria floribunda	7

Supplementary Table S2. Model evaluation from partial AUC after 500 bootstrapping simulations.

Model	Number of testing points	Mean AUC ratio	*t*	df	*P*-value
Xyleborus glabratus (Bioclimatic)	198	1.735306	1069.7	524.38	< 2.2e-16
Xyleborus glabratus (NDVI)	198	1.417213	214.64	678.62	< 2.2e-16
Euwallacea sp. (Bioclimatic)	423	1.559872	319.7	589.32	< 2.2e-16
Euwallacea sp. (NDVI)	423	1.970992	3607	500.27	< 2.2e-16
Hosts *Euwallacea* sp. (NDVI)	1251				
Lauraceae species (NDVI)	2262	1.625564	392.18	550.21	< 2.2e-16
Xyleborus species (NDVI)	517	1.251977	23.296	794.12	< 2.2e-16
